# Plasmidome-Analysis of ESBL-Producing *Escherichia coli* Using Conventional Typing and High-Throughput Sequencing

**DOI:** 10.1371/journal.pone.0065793

**Published:** 2013-06-13

**Authors:** Alma Brolund, Oscar Franzén, Öjar Melefors, Karin Tegmark-Wisell, Linus Sandegren

**Affiliations:** 1 Swedish Institute for Communicable Disease Control, Solna, Sweden; 2 Department of Microbiology, Tumor and Cell biology, Karolinska Institutet, Stockholm, Sweden; 3 Department of Cell and Molecular Biology, Karolinska Institutet, Stockholm, Sweden; 4 Department of Medical Biochemistry and Microbiology, Uppsala University, Uppsala, Sweden; Institut National de la Recherche Agronomique, France

## Abstract

Infections caused by Extended spectrum β-lactamase (ESBL)-producing *E. coli* are an emerging global problem, threatening the effectiveness of the extensively used β-lactam antibiotics. ESBL dissemination is facilitated by plasmids, transposons, and other mobile elements. We have characterized the plasmid content of ESBL-producing *E. coli* from human urinary tract infections. Ten diverse isolates were selected; they had unrelated pulsed-field gel electrophoresis (PFGE) types (<90% similarity), were from geographically dispersed locations and had diverging antibiotic resistance profiles. Three isolates belonged to the globally disseminated sequence type ST131. ESBL-genes of the CTX-M-1 and CTX-M-9 phylogroups were identified in all ten isolates. The plasmid content (plasmidome) of each strain was analyzed using a combination of molecular methods and high-throughput sequencing. Hidden Markov Model-based analysis of unassembled sequencing reads was used to analyze the genetic diversity of the plasmid samples and to detect resistance genes. Each isolate contained between two and eight distinct plasmids, and at least 22 large plasmids were identified overall. The plasmids were variants of pUTI89, pKF3-70, pEK499, pKF3-140, pKF3-70, p1ESCUM, pEK204, pHK17a, p083CORR, R64, pLF82, pSFO157, and R721. In addition, small cryptic high copy-number plasmids were frequent, containing one to seven open reading frames per plasmid. Three clustered groups of such small cryptic plasmids could be distinguished based on sequence similarity. Extrachromosomal prophages were found in three isolates. Two of them resembled the *E. coli* P1 phage and one was previously unknown. The present study confirms plasmid multiplicity in multi-resistant *E. coli.* We conclude that high-throughput sequencing successfully provides information on the extrachromosomal gene content and can be used to generate a genetic fingerprint of possible use in epidemiology. This could be a valuable tool for tracing plasmids in outbreaks.

## Introduction

Extended spectrum β-lactamases (ESBLs) are bacterial enzymes that catalyze hydrolysis of β-lactam antibiotics with extended spectrum (e.g., penicillins and cephalosporins) [1], and undermine the present widespread clinical use of these antimicrobial agents. ESBLs are frequently found in commensal bacteria of the gut microbiota of humans and animals, such as *Escherichia coli*, that are exposed to antimicrobial agents ingested by the host. As an extraintestinal pathogen, *E. coli* frequently causes urinary tract infections, bacteraemia, and neonatal meningitis. Individuals can contract ESBL-producing *E. coli* from the community or in hospitals, and the prevalence of these organisms can reach up to 80% in certain parts of the world [2]. In Europe, resistance rates to 3^rd^ generation cephalosporins among invasive *E. coli* isolates ranges from 3 to 36% with Sweden and Norway having the lowest numbers (http://ecdc.europa.eu/en/activities/surveillance/EARS-Net/).

ESBLs are heterogeneous enzymes that are categorized based on structure and function [3]–[5]. CTX-M enzymes are the most widespread ESBLs, and appear to have originated from genomic CTX-M-like genes from non-pathogenic environmental *Kluyvera* spp. [6]. Mobilisation of these genes via transposons onto plasmids has led to the successful dissemination and adaptation of CTX-M enzymes in pathogenic bacterial clones [6]. At least 130 CTX-M variants are described to date, and these can be divided into five phylogroups based on amino acid identities: CTX-M-1, CTX-M-2, CTX-M-8, CTX-M-9, and CTX-M-25. The CTX-M-15 enzyme (belonging to phylogroup 1) is the most common type and has spread globally. Interestingly, *in vitro* studies have shown that the full diversity of ESBL enzymes has not been reached [7], implying that new variants of these enzymes are yet to emerge.

Plasmids are autonomously replicating extrachromosomal elements (replicons) that often contain the genes needed for their own replication and horizontal transfer, and accessory genes such as those for antibiotics resistance. ESBL-genes are encoded on large plasmids, often together with genes conferring resistance to other antimicrobial classes [6]. As a result, plasmids are sustained in the host by conferring a selective advantage in the presence of antibiotics. Furthermore, plasmids play a central role in dispersion of resistance determinants across different taxonomic and ecological bacterial groups [8], [9]. Several plasmid types have been specifically associated with ESBL-genes [6]: *bla_CTX-M-15_* is mostly associated with IncFII plasmids; IncN, IncI1, and IncL/M plasmids are associated with various *bla*_CTX-M_; and IncK plasmids are associated with *bla*_CTX-M-14_. Despite these relationships, there is no certain association between replicon-type and *bla*_CTX-M_-type [10].

Conventional methods for plasmid typing (e.g., electrophoretic plasmid-profiling, PCR, and Southern blot) have low discriminatory power. Sequence-based methods have the potential to overcome this limitation by providing an overview of the complete gene content. Nevertheless, clone-by-clone sequencing of individual plasmids is a time-consuming task. The plasticity of plasmids is problematic for typing of these molecules, since the plasmid structure can vary between studies. The latter complicates interpretation of data from methods like pRFLP (plasmid restriction fragment length polymorphism). This typing tool is best suitable for plasmids isolated closely in time, e.g., during an ongoing outbreak. In this study we have analyzed the plasmidomes (the complete plasmid content) of ten diverse clinical *E. coli* isolates using high-throughput sequencing combined with conventional molecular methods. Our aims were to improve our understanding of the plasmid composition in ESBL-producing *E. coli* and to evaluate the usefulness of high-throughput sequencing as a tool for metagenomics-like plasmid analysis. The approach presented in this study could also be useful for epidemiological typing of plasmidomes. Our study indicated high genetic diversity of the analyzed plasmidomes, and an abundance of small plasmids, most of which were of unknown function.

## Materials and Methods

### Accession Numbers

Roche 454 pyrosequencing reads have been deposited in NCBI’s Short Read Archive under the accession number SRA052673. Assembled contigs representing complete plasmids have been deposited in NCBI’s GenBank under accession numbers: JX238440 (pEC08-1), JX238441 (pEC08-2), JX238442 (pEC08-3), JX238443 (pEC08-4), JX238444 (pEC08-5), JX238445 (pEC08-6), JX238446 (pEC19-1), JX238447 (pEC29-1), JX238448 (pEC33-1), JX238449 (pEC71-1), JX238450 (pEC71-2), JX238451 (pEC135-1), JX238452 (pEC147-1), JX238453 (pEC147-2), JX238454 (pEC147-3), JX238455 (pEC147-4), JX238456 (pEC163-1), JX238457 (pEC299-1), JX238458 (pEC299-2), JX238459 (pEC299-3), and JX238460 (pEC299-4).

### Sample Preparation and Characterization

Ten diverse ESBL-producing *E. coli* isolates were selected from a large Swedish collection previously described [11]. The isolates have been subjected to antibiotic susceptibility testing, real-time PCR for *bla*_CTX-M_ phylogrouping, pulsed field gel electrophoresis (PFGE), and multilocus sequence typing (MLST) [11].

S1 nuclease digestion analyzed with PFGE, and Southern blot were performed as previously described [12]. Southern blot probes were generated by DIG labelling of PCR products using the DIG DNA Labelling Kit (Roche Applied Sciences, Penzberg, Germany). Probes detecting replicons were obtained from the PBRT protocol [13], and for *bla*_CTX-M_ detection using general primers from [14]. Plasmid sizes were estimated using BioNumerics v.6.6 by comparison to a PFGE low range marker (New England Biolabs). The S1/PFGE analysis was repeated four times for all isolates and the mean value of the size was used for each plasmid. Capillary Sanger sequencing was performed to determine the *bla*_CTX-M_ genotype.

The plasmid content in each of the ten *E. coli* isolates was extracted and subjected to high-throughput sequencing. The isolates were grown on blood agar overnight with antibiotics selection (cefotaxime). Liquid cultures were then grown in Luria-Bertani broth, without antibiotic selection. Plasmid extractions were performed using the Qiagen MIDI plasmid purification kit according to the manufacturer’s instructions. PCR-based replicon typing (PBRT) was performed as previously described [13]. Sequence typing of IncF replicons and plasmid MLST of IncI1 plasmids were performed [15], [16] by analysis of Roche 454 pyrosequencing reads (see below). Plasmid sequence types were analyzed using the Plasmid MLST Databases (http://pubmlst.org/plasmid/) [17]. Antibiotic resistance categorization was determined by following EUCAST guidelines (http://www.eucast.org/clinical_breakpoints/).

### Shotgun Sequencing, Sequence Assembly, and Bioinformatic Analysis

High-throughput sequencing was performed using the Roche 454 GS FLX instrument. To generate libraries that were individually tagged using ten MID adaptors (Roche Diagnostics), 5 µg of RNA-free DNA was used. Libraries were subsequently pooled, subjected to emulsion PCR, and sequenced on a two-region picotiter plate. All procedures, including the ensuing sequence acquisition and processing, followed the standard protocol of the manufacturer (v.1.1.03). The amount of chromosomal DNA was estimated by mapping sequencing reads against the *E. coli* chromosome (U00096.2) using the alignment program bwa v.0.6.1 [18] using the BWA-SW component. Workflow for analysis of the plasmid content: *de novo* assembly was performed for each data-set with Newbler v.1.1.03 (Roche) and the CLC Genomics workbench v.6 (CLC Bio, Aarhus, Denmark). Contigs were subjected to BLASTn analysis against the GenBank nt database (NCBI) and organized according to best matching reference sequence. Reference plasmid sequences that attracted the majority of BLASTn hits were used for reference assembly using CLC Genomic Workbench with minimum similarity matching parameters set to 0.8. Reference assemblies of the best matches were analyzed for single nucleotide polymorphisms, small deletion/insertion polymorphisms, and rearrangements using the respective analysis tool in CLC Genomics Workbench. To verify that no plasmids were missed, *de novo* assembly of non-matched reads from combined reference assemblies for each dataset was performed and remaining contigs were subjected to BLASTn searches and analysed for gene content.

### Metagenomic Analysis of Sequencing Reads

Scripts were written in Perl v.5.12.4 and R v.2.14.1 (http://www.r-project.org/), and are available on request. *Domain analysis:* The complete Pfam-A v.26.0 database [19] was retrieved locally (ftp://ftp.sanger.ac.uk/pub/databases/Pfam/releases/Pfam26.0/), and the database was formatted using the ‘hmmpress’ command in HMMER v.3.0 (default settings) [20]. Pfam-B models were ignored since these are of lower quality. Roche 454-pyrosequencing reads were conceptually translated to all six reading frames with a BioPerl script. Sequences were then queried against the HMM database with the command ‘hmmscan’ (default settings). A Gentoo Linux (kernel v.3.0.0) system (specifications: x86_64 Intel Xeon CPU X7550, 2.00GHz) was used for the HMMER execution, consuming about 142 CPU hours. The output data were parsed with a Perl script. Domain hits were considered significant if the E-value <1e–05; the best hit for each sequencing read was considered to be the correct one. For each sample, the number of reads with domain-hits was normalized with the total number of reads for the sample, multiplied by 10^5^, and log_10_-transformed (values were incremented by 1 to avoid infinite numbers). The domain distribution was then visualized using the heatmap.2 function of the gplots R-package (http://cran.r-project.org/web/packages/gplots/). *Resistance gene analysis:* A database consisting of 150 sequences of known resistance genes was established. Accession numbers are found in Table S1. Sequences were manually grouped into 35 gene families based on function and homology. Multiple alignments of amino acid sequences were then created of sequences of the same group using the software ClustalW v.2.1 (default settings) [21]. Columns in the multiple alignment containing gaps were removed with a Perl script. Next, profile Hidden Markov Models (pHMM) were built from the alignments using the ‘hmmbuild’ command of HMMER (default settings). pHMMs were compressed to a single database using ‘hmmpress’ (default settings). 454-reads were translated into the six reading frames. The database was searched using ‘hmmscan’. Criteria for positive hits were E-value ≥1e–20, query coverage ≥85% of the reference alignment used to create the pHMM and the sequence identity ≥92%. The binary outcome of each HMM was adapted into a matrix where columns represented groups and rows represented samples. The matrix was then visualized as described above.

## Results and Discussion

### Characteristics of the Selected *E. coli* Isolates

**Figure 1:**
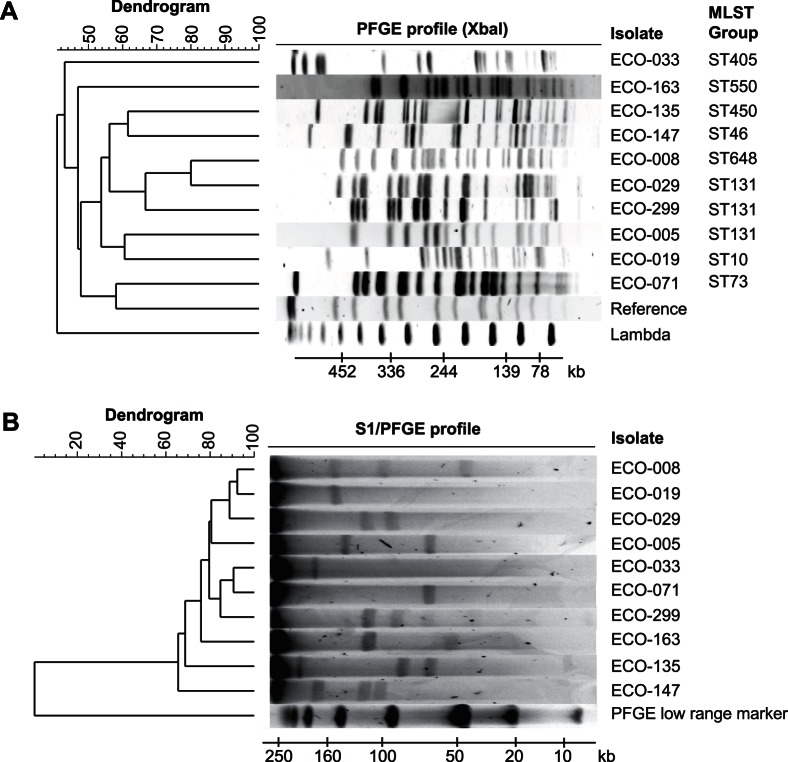
Epidemiologic strain typing and S1/PFGE plasmid profiles. (A) Total *E. coli* DNA digested with the restriction enzyme Xbal and analysed using pulse-field gel electrophoresis (PFGE). Fingerprints were analysed using BioNumerics v. 6.6. The name of the isolate and the multilocus sequence type (MLST) is indicated to the right (under ‘MLST Group’). The dendrogram to the left shows sample similarity by UPGMA clustering of PFGE patterns. The bottom scale-bar refers to size of PFGE bands in kilobases. (B) S1/PFGE analysis of extrachromosomal DNA (e.g., plasmid and phage DNA) of the ten isolates.

The isolates described in this study were originally sampled from patients with urinary tract infections [11]. The isolates were selected based on the following criteria: (*i*) diverse PFGE (<90% similarity with PFGE); (*ii*) diverse geographical origins within Sweden; and (*iii*) divergent antibiotic susceptibility profiles. The PFGE results are shown in Figure 1A. Sensitivity to 13 antibiotics was tested using disk diffusion test and Etest (Table 1). The ten isolates were all categorized as sensitive to mecillinam, amikacin, and nitrofurantoin, while resistant to cefadroxil and cefotaxime. Notably, one isolate (ECO-008) was resistant to 8 of 13 tested antibiotics, and represented the most resistant isolate of the study. In addition to PFGE, *E. coli* multilocus sequence typing (MLST; Figure 1A), identified eight sequence types (STs). Three isolates belonged to the globally disseminated ST131 [22]. A survey of the literature found that 6 of 8 identified STs (405, 46, 648, 131, 10, and 73) have previously been associated with urinary tract infections. The ten isolates originated from eight geographically dispersed microbiological laboratories in Sweden. Isolates ECO-005 and 008 originated from the same laboratory, this was also the case for ECO-029 and 033. However, the isolates were of distinct PFGE- and ST-types, indicating that they were unrelated.

**Table 1:** Plasmid characteristics and content.

	Conventional typing				Bioinformatic results		
Isolate (ECO-)	Susceptibility test	Plasmid size (kb)	Replicon type	CTX-M phylogroup	Best match	Resistance genes	Replicon sequence types
005	R: CDR, CTX, NAL, GEN, TMPI: CAZ, CFP	139	F, FIB	nd	pUTI89 (CP000244) 88% covered, 101 kb (12x), 99.3% identity	nd	F29:A-:B10
		67	F	CTX-M-9	pKF3-70 (FJ494913) 95% covered, 63 kb (18x), 99.3% identity	*bla_CTX-M-14_*	F35:A-:B-
					8 contigs >1 kbp, ∑ 28 kb (6–28x)	*aac(3)III, aadA5, bla_TEM-1,_ dfrA7, mphR, mrx, mph(A), strA+B, sul1, sul2, tetA*	nd
008	R: CDR, CTX, CAZ, CFP, PTZ, NAL, CIP, GEN, TMPI: TOB	151	F, FIB	CTX-M-1	na	na	na
		103	nd	nd	na	na	na
		42	nd	nd	Phage, no match, 47 kb (12x)	nd	nd
					15 contigs >1 kbp, ∑ 19 kb (4x)	*Partial coverage: aadA5, bla_TEM-1_, dfrA7, mphR, mrx, mph(A), sul1*	F1:A-:B1 (partial coverage)
019	R: CDR, CTX, CAZ, NAL, CIP, GEN, TOB, TMPI: CFP, PTZ	150	F, FIA, FIB	CTX-M-1	pEK499 (EU935739) 80% covered, 98 kb (17x), 99.5% identity	*aac(6′)-Ib-cr, aadA5, aph(3′), bla_CTX-M-15_, bla_OXA-1_, bla_TEM-1_, catB3, dfrA7, mphR, mrx, mph(A), sul1, tetA*	F1:A1:B1
					11 contigs >1 kbp, ∑ 46 kb (14–52x)	*aac(3′)-II, strA+B, sul2, tetRACD*	nd
029	R: CDR, CTX, PTZ, NAL, CIP, TMPI: CAZ, CFP, TOB	124	F, FIA	CTX-M-1	pEK499 (EU935739) 69% covered, 85 kb (35x), 99.99% identity	*aac(6′)-1b-cr, aadA5, bla_CTX-M-15_, bla_OXA-1_, catB4, dfrA7, mphR, mrx, mph(A), sul1*	F2:A1:B-
		96	F, FIA	CTX-M-1	P1-like phage 81% covered, 89 kb (39x), 98.0% identity	nd	nd
					6 contigs >1 kbp, ∑ 13 kb (21–28x)	nd	nd
033	R: CDR, CTX, CFP, NAL, CIP, GEN, TMPI: CAZ, PTZ, TOB	182	F, FIA, FIB	CTX-M-9	pKF3-140 (FJ876827) 96% covered, 142 kb (8x), 99.9% identity	nd	F1:A6:B20
					12 contigs >1 kbp, ∑ 30 kb (5–14x)	*aac(3′)II, aac(6′), aadA5, bla_CTX-M-14_, bla_TEM-1_, cmlA7-like, dfrA7, ermB+C, mphR, mrx, mph(A)*, *strA+B*, *sul1*, *sul2*, *tetA*	nd
071	R: CDR, CTXI: CAZ, CFP, PTZ	68	F	CTX-M-9	pKF3-70 (FJ494913) 99% covered, 69 kb (52x), 99.9% identity	*bla_CTX-M-14_*	F2:A-:B-
					No contigs >1 kbp	na	na
135	R: CDR, CTX, NAL, CIP, GEN, TOB, TMPI: CAZ, CFP	213	F, FIB	nd	p1ESCUM (CU928148) 93% covered, 113 kb (6x), 99.9% identity	nd	F29:A-:B10
		87	I1	CTX-M-9	pEK204 (EU935740) 90% covered, 84 kb (21x), 98.4% identity	nd	IncI1 ST105
		67	F	CTX-M-9	pHK17a (JF779678) 98% covered, 68 kb (33x), 98.4% identity	*bla_CTX-M-14_*	F53:A-:B-
					17 contigs >1 kbp, ∑ 30 kb (3–34x)	*aac(3′)II*, *aac(6′)-Ib*, *aadA5*, *cmlA1*, *dfrA7*, *mphR*, *mrx*, *mph(A)*, *strA+B*, *sul1*, *sul2*, *tetA*	nd
147	R: CDR, CTX, NAL, CIP, TMPI: CAZ, CFP, GEN	181	F, FIA, FIB	nd	p083CORR (CP001856) 86% covered, 128 kb (8x), 99.2% identity	*bla_TEM-1_*, *catA1*, mer-operon, *strA+B*, *sul2*, *tetA*	F31:A4:B1
		121	I1	CTX-M-9	R64 (AP005147) 86% covered, 103 kb (16x), 98.6% identity	ars-operon, *strA+B*	IncI1 ST71
		107	nd	nd	pLF82 (CU638872) 75% covered, 81 kb (12x), 97.5% identity	nd	nd
					31 contigs >1 kbp, ∑ 87 kb (5–23x)	*aph(3′), bla_CTX-M-65_, fosA3*	nd
163	R: CRD, CTX, CFPI: CAZ	121	F, FIA, FIB	nd	pSFO157 (AF401292) 54% covered, 69 kb (22x), 98.0% identity	nd	F60:A3(trunk 137 bp/408):B31
		55	nd	CTX-M-1	R721 (AP002527) 63% covered, 48 kb (22x), 98.3% identity	nd	nd
					14 contigs >1 kbp, ∑ 59 kb (13–35x)	*bla_CTX-M-1_*	nd
299	R: CDR, CTX, NAL, CIP, TMPI: CAZ, CFP, PTZ, GEN, TOB	121	F, FIA	CTX-M-1	pEK499 (EU935739) 80% covered, 97 kb (8x), 99.9% identity	*aadA5*, *bla_TEM-1_*, *dfrA7*, *mphR*, *mrx*, *mph(A)*, *sul1*	F2:A1:B-
		95	Y	nd	P1-like phage 82% covered, 77 kb (5x), 98.1% identity	nd	Y
		19	nd	nd			
		8	nd	nd	no match, 9 kb (33x)	nd	nd
					8 contigs >1 kbp, ∑ 38 kb (6–10x)	nd	nd

a Average 454 coverage is specified within parenthesis.

Abbreviations: (nd) not detected; (na) not applicable; and ∑ sum of unassigned contigs.

### Plasmids of the Selected *E. coli* Isolates

Total DNA of each isolate was analyzed with S1/PFGE. This method is used to determine the number and sizes of plasmids in each bacterium. The procedure indicated at least 22 putative plasmids with an estimated size between 8–213 kb (Figure 1B). The number of large plasmids per isolate ranged from one to four. Sherley *et al.* reported *E. coli* to contain in average 2.5 different plasmids per cell [23], and the samples thus had a plasmid count within the expected. There was no correlation between number of plasmids and antibiotic sensitivity (Spearman’s rho = 0.050, *p* = 0.88), suggesting that several resistance determinants are encoded on the same replicon. There was no indication of plasmids smaller than 8 kb, consistent with the known limitation of S1/PFGE to resolve very small plasmids. However, plasmids smaller than 8 kb were identified in nine of ten isolates on regular 2% agarose-gel electrophoresis (not treated with S1 nuclease). However, their exact numbers and sizes were not determined (data not shown). CTX-M phylogroups were determined using real-time quantitative PCR (qPCR) [24], and Southern blot was used to link CTX-M phylogroups and replicon types to specific plasmids on the S1/PFGE gel (Table 1). Phylogroups CTX-M-1 or CTX-M-9 were identified in the isolates, consistent with these groups being the most encountered in Sweden [25].

### Plasmid High-throughput Sequencing Data

Shotgun 454-pyrosequencing of barcoded extracted plasmid DNA generated a median of 29,205 reads/sample with a median read length of 239 bp (Table S2, Figure S1A), consistent with the expected read length of the sequencing chemistry. The amount of reads mapping to chromosomal DNA in the plasmid preparation was estimated to an average of 12.8% (Figure S1B) and the uniform read distribution on the *E. coli* chromosome suggested that the reads were of contaminating chromosomal origin (Figure S1C). Chromosomal reads were excluded from further analysis.

Non-chromosomal sequencing reads were assembled into continuous sequences (contigs) *in silico*. The number of contigs ranged from 112 (ECO-299) to 2,676 (ECO-033). Five samples had N50 values under 1 kb, indicating the presence of many small contigs (Table S2). Subsequently, we explored the plasmid content of the isolates by manual examination of contigs longer than 10 kb by nucleotide BLAST (GenBank). This procedure identified 15 variants of 13 different GenBank-deposited plasmids with reference coverage between 54–99% (average 83%) and a sequence identity in matching regions between 97.5–99.99% (Table 1). Reads that could not be assembled to the best matching reference were *de novo* assembled and contigs >1 kb were analysed. The number of plasmid-variants in each isolate ranged from zero (ECO-008) to three (ECO-135 and 147). Variants of the pEK499 plasmid were identified in three samples (ECO-019, 029, and 299), suggesting that this plasmid has been relatively successful in its dissemination.

### Extrachromosomal Prophages in Three Plasmidome Samples

BLASTn searches of assembly contigs indicated that at least three isolates (ECO-008, 029, and 299) contained extrachromosomal prophages (Table 1). Both ECO-029 and ECO-299 gave extensive matches (89.8 kb and 77 kb, respectively) with 98% sequence identity to the *E. coli* P1 phage (AF234172), which is known to replicate as an extrachromosomal entity [26]. ECO-008 had only one contig (47.2 kb) over 10 kb in length, which did not produce any coherent nucleotide matches to known phages or plasmids. However, this contig contained 13 open reading frames (ORFs) longer than 300 bp, 11 of which produced hits to phage-like protein sequences upon BLASTp-analysis. Hence, the identified contig from ECO-008 likely represents the complete or partial genome of a phage. DNA fragments corresponding to phages in the S1/PFGE-analysis could be assigned (Table 1). In addition, isolate ECO-147 contained one large plasmid matching to pLF82 (CU638872) [27], containing distinct features resembling a phage and could represent an additional extrachromosomal prophage.

Overall, the data demonstrated that at least three out of ten samples contained extrachromosomal prophages, which are easily mistaken for plasmids on S1/PFGE analysis. As a result, S1/PFGE risks overestimating plasmid content. Conversely, prophage activation can bias results from sequencing-based assays. The combination of S1/PFGE and sequencing should therefore be considered in plasmidome characterization.

### Consistency between Molecular Data and Sequencing Data

The results from all methods used for plasmid analysis is concluded in Table 1. While there was a high general agreement between S1/PFGE and the 454 data (e.g., the sum of contigs match well the estimated total size of plasmids from S1/PFGE), some exceptions were found.

S1/PFGE of ECO-008 resulted in DNA fragments at 42, 103, and 151 kb where only the 42 kb fragment could be assigned to a putative phage. No longer contigs corresponding to plasmid sequences were found in the ECO-008 data and the short contigs produced had very low coverage. This indicates that in ECO-008 phage assisted DNA degradation of the large plasmids had occurred in the culture used for the sequencing sample. Whether this was due to spontaneous prophage activation or contamination with a virulent phage is uncertain since the resulting phage sequence has no close match in the databases. However, small plasmids were still found with high sequence coverage in this isolate (see below).

In Figure 1B six bands can be seen on the S1/PFGE gel for ECO-299. 454-data confirmed three of the plasmids with sizes 121, 95 and 8 kb. Two bands approximately 71 kb and 42 kb are also seen on the S1/PFGE gel. The Southern blot results showed the same replicon types and *bla*_CTX-M_ gene to be linked to these to fragments that were also found on the large 121 kb plasmid. Our interpretation of this is that the large 121 kb plasmid has been divided in two either due to excess cleaving of the S1 nuclease or due to variations in the plasmid composition. The large plasmid being a multi-replicon plasmid could make division in two smaller compatible plasmids possible. Presence of the 71 and 42 kb bands also varied upon repetition of the S1/PFGE analysis further supporting our conclusions. Figure 1B also show isolate ECO-299 to have a 19 kb DNA fragment which was not identified in the 454-data. There are several plausible explanations for this, the most likely being that the dynamic nature of plasmids causes inconsistencies on S1/PFGE.

Isolate ECO-029 had a good agreement between S1/PFGE results and the 454-data. However, the Southern blot assigned the replicons F and FIA as well as the *bla*_CTX-M_ gene to both fragments of 124 and 96 kb. We think that this represents false positive signals from the Southern blot probes. Apart from these examples, the numbers and sizes of the large plasmids correlated well with the bioinformatic analysis of the 454-data. In conclusion, the S1/PFGE method was mostly consistent with *in silico* analyses of gene content based on 454-pyrosequencing.

### Small Cryptic Plasmids

**Figure 2:**
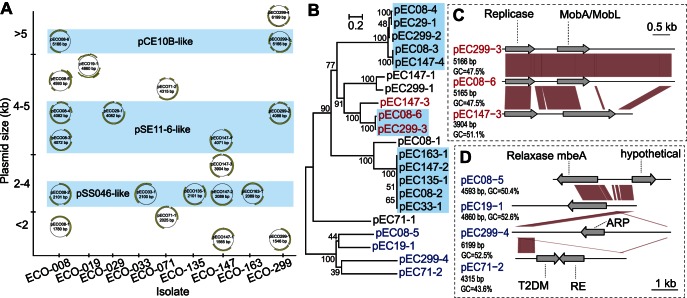
Distribution and properties of small cryptic plasmids. (A) Illustration of the content of small cryptic plasmids in the isolates. Circles represent plasmids and the plasmid size is shown on the y-axis in kilobases and the x-axis shows the sample ECO- identifier. (B) Neighbour-joining tree of small cryptic plasmids indicating their sequence-based relationships. Sequences were aligned with ClustalW v.2.1 and the tree was created with MEGA v.5 (default settings) [35]. Scale bar = substitutions per site. Support values are indicated at branches. (C and D) Sequence similarity blocks between small cryptic plasmids (violet stripes). Arrows indicate ORFs longer than 300 bp. Similarity blocks were identified with Mauve v.2.3.1 [36], and plotted with genoPlotR v.0.8.1. [37]. ARP = putative aminoglycoside resistance protein; T2DM = putative type II DNA- methyltransferase; RE = putative EcoVIII restriction endonuclease.

Multiple small plasmids (<8 kb) were found in all isolates except ECO-005. These plasmids were in the size range 1.5 to 6.2 kb, displayed %GC between 43.6 to 52.6% (median = 49.7%), and will be referred to as small cryptic plasmids (SCPs). SCPs were identified as assembly contigs with deep coverage, containing discriminatory circularization signatures at contig ends. All but one SCP matched plasmids of similar sizes in GenBank with high significance, corroborating the idea that these contigs represent complete plasmids rather than being fragments of larger plasmids. Five isolates contained one SCP, while four isolates contained 6, 2, 4, and 3 different SCPs, respectively (Figure 2A; Table S3). SCPs were given names reflecting the isolates they were found in. The 2.0 kb plasmid pEC71-1 (smallest plasmid from ECO-071) displayed 52.2% GC, contained a single 990-bp open reading frame (ORF), and did not produce any significant hits in GenBank. All other SCPs had >96% sequence identity with plasmids of similar size (Figure 2AB). Most cryptic plasmids only contained genes for replication and plasmid mobility.

Interestingly, several of the SCPs were highly similar between isolates, although the isolates had intentionally been selected to be as diverse as possible. SCPs could be grouped into three clustered groups based on sequence similarity (Figure 2A and B). The most ubiquitous and conserved were pSS046-like plasmids, found in five isolates. Plasmid pEC08-2 was identical to the pSS046 plasmid sequence in GenBank while the other four differed by one to twelve base substitutions or base deletions. Similarly, NA114-like and pSE11-6-like plasmids were found in isolates ECO-008, 029, 147 and 299; and pCE10B-like plasmids in isolates ECO-008 and 299. Interestingly, pEC299-4 only contained a single detectable open reading frame, likely encoding an aminoglycoside resistance protein and pEC71−2 contained only two open reading frames oriented tail to tail, encoding a putative type II DNA-methyltransferase and a putative EcoVIII restriction endonuclease. Illustrations of selected SCPs and their gene content is shown in Figures 2C and D.

In conclusion, SCPs appeared in almost all isolates, suggesting that these are common in ESBL-producing *E. coli*. The latter is corroborated by previous observations [28]. We find cross-contamination to be unlikely, since in that case one would expect perfect sequence similarity between samples. It remains unclear if SCPs confer any selective advantage to bacteria, or if these elements simply exists as very successful selfish genetic elements.

### Plasmid Content of the ST131 Samples

Three of the ten isolates (ECO-005, 029, and 299) belong to the *E. coli* lineage ST131. Isolates ECO-029 and 299 contained plasmids with high sequence similarity (>99.9%) to the previously sequenced ST131 ESBL-plasmid pEK499 (EU935739) [29], but with matches to only 69 and 80% of the pEK499 sequence, respectively, due to larger rearrangements. Most notable was the lack of tra-genes in the ECO-029 sequence. The two isolates had almost identical resistance profiles (Table 1). Both isolates also contained a P1-like prophage. A pEK499-like plasmid was also found in ECO-019, belonging to ST10, indicating that this plasmid type is not restricted to the ST131 lineage. ECO-005 also belongs to the ST131 lineage but contained two different plasmids matching pUTI89 (CP000244) and pKF3-70 (FJ494913). The former is a 114.2 kb plasmid occurring in uropathogenic *E. coli* [30] whereas the latter is a 70.0 kb plasmid common in *Klebsiella pneumoniae* [31]. In addition, ECO-005 did not contain any small cryptic plasmids while ECO-029 and ECO-299 both contained very similar versions of pSE11-6-like plasmids. ECO-299 contained four additional small cryptic plasmids (1,546 to 8,826 bp) that were not found in any of the other ST131 isolates but had counterparts in isolates not belonging to this group. Analyses of the plasmid content of ST131 isolates has been reported before [32], and found to vary. However, no reports have been published on small cryptic plasmids in ST131. Our results illustrate the highly dynamic plasmid content of this widespread and successful group of *E. coli*.

### Detection of Resistance Genes

**Figure 3:**
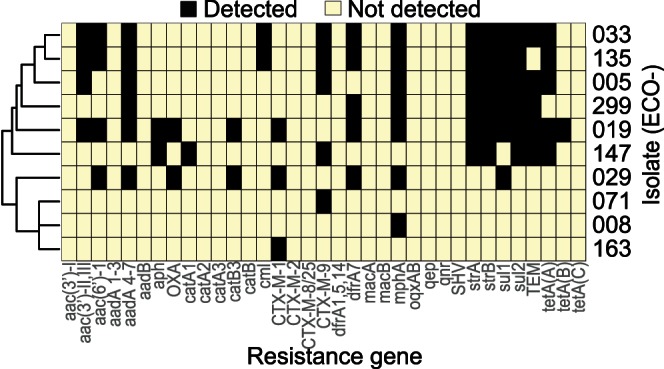
Resistance genes in the plasmidomes. Heat-map analysis of resistance genes. Searches were performed on unassembled 454-pyrosequencing reads using 35 custom profile Hidden Markov Models. The gene family is shown on the x-axis and the isolate identifier is shown on the y-axis (ECO-). Black indicates that the gene was found. Employed criteria to define a gene as found: E-value = 1e-20; percent identity>92%; and percent HMM query coverage >85%.

454-pyrosequencing data were used to identify genes conferring resistance to the following groups of antibiotics: β-lactams, fluoroquinolones, trimethoprim, sulphonamides, macrolides, aminoglycosides, tetracycline, and chloramphenicol. Overall, 19 of the 35 gene families included in the pHMM database were identified in the data, and the distribution is shown in Figure 3. In two isolates (ECO-008 and 299) we did not detect the *bla*_CTX-M_ known to exist from the molecular methods. For ECO-008 this is likely explained by the fact that the plasmid DNA was, to a large extent, phage degraded as discussed above and did not produce hits that matched the pHMM criteria. In ECO-299, we found that the pEK499-like plasmid had an 18 kb deletion between two directly repeated IS26 elements flanking resistance genes, including the *bla*_CTX-M-15_ gene. The *bla*_CTX-M_ gene was shown to be plasmid mediated in the S1/PFGE/Southern blot analysis. The culture conditions were the same in preparation for S1/PFGE as well as preparation of whole genome DNA for 454-sequencing. IS26 elements are commonly associated with resistance regions and promote homologous recombination leading to genetic rearrangements [33]. This truly reflects the dynamic nature of plasmids and illustrates that the sequence of a plasmid is a snapshot of the plasmid conformation at the time of analysis.

Three isolates (ECO-008, 071, and 163) were found to have only one resistance gene. For ECO-071 and ECO-163 this was confirmed in the antibiotic susceptibility testing (Table 1) while in ECO-008 it was again an effect of the low sequence coverage. In the remaining isolates we identified eight to fifteen additional resistance determinants. Upon examination of the genomic environment where the gene was situated, we frequently identified class 1 or class 2 integrons. In six of the isolates (ECO-005, 019, 033, 135, 147, and 163) resistance genes not present in the best matching reference plasmid were found in additional contigs, reflecting the dynamic nature of these genes. These genes are listed among extra contig sequences in Table 1. The majority of resistance genes were encoded on large plasmids. Only one of the small cryptic plasmids contained resistance genes, the 6.2 kb p62-like plasmid pEC299-4 that contained *sul2* and *strAB* genes.

### Identification of ESBL- and Replicon Types

PCR based protocols for the detection of ESBL-phylotypes are abundant and give information on what class of gene is present but DNA sequencing is usually required for the exact genotype. PCR based replicon typing (PBRT) is one of the most common methods for plasmid typing, but provides limited resolution to profile samples containing multiple replicons. PBRT also have a relatively low discriminatory power. In addition, none of the PCR based methods can link the target gene to a specific plasmid. Sanger sequencing and 454-sequencing data were used to identify CTX-M alleles and replicon types and S1/PFGE followed by Southern blotting mapped them to the respective plasmid. Plasmids harbouring *bla*_CTX-M_ were between 55 to 182 kb in size and plasmids of the IncF-type were the most common. Two plasmids were of incompatibility group I1 and associated to genes of the CTX-M-9 phylogroup. Interestingly, plasmids in ECO-033, 147, and 163 were found to encode ESBL-genes not present in their best matching GenBank reference plasmids in spite of otherwise generally high sequence identity. Thus, it suggests that *bla*_CTX-M_ genes have been acquired by plasmids normally circulating in the *E. coli* population, rather than clonal spread of *bla*_CTX-M_-containing plasmids. Bengtsson *et al.* has showed that plasmids of the same replicon types as found in resistant isolates were present in a collection of antibiotic sensitive *E. coli* isolates although at a much lower frequency [34].

As found in this study and others, most *E. coli* plasmids with *bla*_CTX-M_ are of incompatibility group F [10]. Better resolution is however obtained when subtyping within replicon type. To date there are subtyping protocols for five incompatibility groups: IncF, IncI1, IncN, IncHI1, and IncHI2. Subtyping of IncF plasmids results in a FAB-formula, given by the presence and/or type of F, FIA and FIB replicons. Isolate ECO-163 contained one plasmid with a previously unknown IncF FAB-formula, given new allele numbers: FII60 and FIB31. In addition, the IncI1 plasmid in ECO-135 had two novel MLST alleles: ardA19 and pilL13, and were assigned the identifier ST105 (deposited to the plasmid MLST database and GenBank). Plasmids with identical FAB-formula were found in ECO-005 and ECO-135, and in ECO-029 and ECO-299 (the latter two belonging to ST131). The overall large variability is in accordance with previous studies [10], where 67 different FAB-formula were detected in 128 IncF plasmids. In one of the *bla*_CTX-M_-containing plasmids (in ECO-163) it was not possible to detect any replicon with the applied methods.

### Analysis of Total Plasmid Gene-content

**Figure 4:**
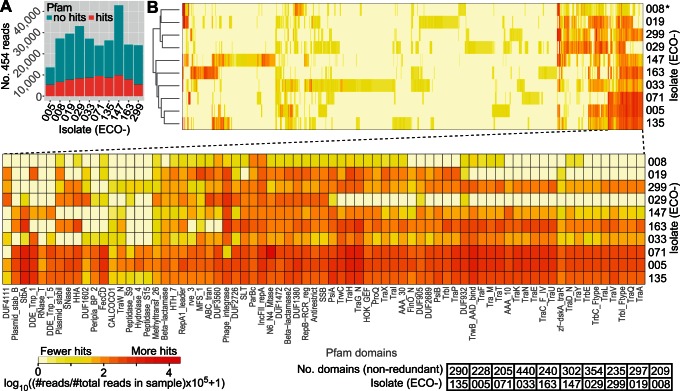
Domain analysis of sequencing reads using pfam profile Hidden Markov Models. (A) Stacked bar-plot showing the number of 454 sequencing reads with hits to pfam models (E-value<1e–05). Turquoise and red colours refer to no hits and hits, respectively. Sequencing reads were conceptually translated to all six reading frames prior to searching. (B) Heat-map analysis of the domain content in the isolates (942 domains were found). The horizontal axis shows the pfam domain and the y-axis indicates the isolate. The unique pfam domain identifier is given on the horizontal axis. The colour intensity is logarithmically related to the number of sequencing reads matching the domain. The white colour indicates few reads matching a domain and colour transition to red indicates that more reads were found. Isolates are clustered after similarity. The bottom-right table indicates the number of non-redundant pfam domains per isolate; i.e., counting each domain only once. (*) The sample has likely been affected by phage-mediated DNA-degradation.

To gain further insight into the protein-coding content of the *E. coli* plasmidome, we performed a heat-map analysis using Pfam conserved protein domains [19] of sequencing reads without sequence assembly (Figure 4A). Analysis of individual sequencing reads can provide domain signatures that are not evident from analysis of contigs. This approach could also be useful for epidemiological typing of plasmids since it creates fingerprints of the plasmidomes. The number of detected domains ranged from 209 (ECO-008) to 440 (ECO-033) (Figure 4B). See Figure S2 and Table S4 for detailed information of all pfam hits found in the supplementary material. The low number of detected domains in ECO-008 likely reflects the phage-mediated degradation of plasmid-DNA. Analysis of sequencing reads of ECO-008 indicated that plasmid domains could indeed be detected, but domain-signals resulting from pfam-models were substantially weaker compared to the other isolates and not sufficient to build contigs *in silico*.

Eighteen domains were common to the isolates (Table S5), and may represent a minimal ‘domain content’. This is not surprising since all isolates contained *bla*_CTX-M_ and IncF plasmids. The phage_integrase domain was detected in all isolates and also occurred in the highest frequency with respect to common domains. Among other common domains were beta-lactamase2, PsiA, DNA methyl-transferase, and helix-turn-helix (DNA binding). The Rep_1 domain (rolling circle replication initiator protein) was abundant in ECO-008, 033, 135, 147 and 163. The restriction endonuclease domain RE_HindIII was found present in the ECO-071 sample, which also contained a strong signature of the N6_N4_Mtase (DNA methylase domain). Notably, there was an absence of F-pilus-related sequences in ECO-008, 019, and 029, suggesting that these isolates have compromised capability to conduct conjugation. This was also corroborated by the absence of reads matching most tra-genes of the best reference hits for these isolates. These results demonstrate the function of gene products in the plasmidome in plasmid mobility, replication, transposition, and possibly defence.

Interestingly, the ECO-071 isolate had the most sensitive antibiotics profile (Table 1), and the domain analysis indicated the lowest domain content (n = 299). The largest number of domains was found in ECO-033 (n = 603), and this isolate was resistant to seven antibiotics. Altogether, there was no clear relationship between number of identified domains and extent of resistance phenotype, indicating that the ‘resistome’ component of the plasmidome occupies one small fraction of the protein-coding potential. Our analysis of the *E. coli* plasmidome shows signatures of abundant genes involved in other processes than strict resistance. This may explain why the loss of resistance is at best a slow process in nature; i.e., multiple resistance determinants are encoded on the same plasmid together with genes encoding other features, and this may provide a selective advantage for the bacterium and assures the survival of the resistance markers.

### Concluding Remarks

This study confirms plasmid multiplicity in clinical *E. coli* isolates. We analyzed the plasmidomes of ten multidrug-resistant *E. coli* samples, and identified two to eight distinct plasmids per isolate. All but the smallest cryptic plasmid were found to be variants of plasmids described in the literature; i.e., there was no evidence of completely novel plasmids. However, compared to the reference sequences deposited in GenBank, plasmids detected in our isolates contained multiple rearrangements, deletions and insertions, mainly among resistance genes. The full spectrum of structural variation could not be resolved and was beyond the scope of this study. One exception was the discovery of multiple unusually small, cryptic plasmids. The small cryptic plasmids encoded few proteins and it is unclear whether they confer any selective advantage to the bacterium or simply exist as successful selfish genetic elements. The existence of small cryptic plasmids without any obvious resulting phenotype represents an unsolved problem in plasmid biology and deserves further attention.

In order to circumvent biases introduced during sequence assembly, we also explored the gene content using metagenomic-like analysis of unassembled reads based on profile Hidden Markov Models presented as a heat-map. Analysis of unassembled reads provided information on several valuable characteristics, such as the presence or absence of certain gene families. The main drawback of using profile Hidden Markov Models relates to the steep computational cost. Hence, profile Hidden Markov Models may be of limited value for larger data sets, which may benefit from longer read lengths or reducing sequence redundancy. Information from these analyses may prove useful for the prediction of antibiotic resistance profiles as well as virulence traits of a strain. Although it needs further testing and evaluation, the plasmid heat-map could potentially be used for epidemiological typing of plasmids. This could give important information on plasmid similarity and the dissemination of plasmids e.g., for identification of plasmid outbreaks.

## Supporting Information

Figure S1 **Sequence properties of 454-pyrosequencing data.** (A) Histogram of Roche 454-pyrosequencing read lengths (bin size = 1). Y- and x-axes show the frequency and the read length in base pairs. (B) Stacked bar-plot of 454 sequencing reads mapping to the *E. coli* chromosome. The isolate identifier (ECO-) is shown on the x-axis and number of sequences on the y-axis. Turquoise and red colours refer to plasmid and chromosomal DNA, respectively. (C) Histogram of alignment positions of sequencing reads on the *E. coli* chromosome (bin size = 20,000). The y-axis shows the number of mapped 454 reads and the x-axis shows the position along the chromosome. Colours refer to ECO- isolate identifiers (given to the right).
(EPS)

Figure S2 **High-resolution heat-map of pfam domains.** High-resolution image of Figure 4B.
(EPS)

Table S1 Protein-coding sequences used to create profile Hidden Markov Models. GenBank accession numbers of sequences used to create profile Hidden Markov Models for scanning raw sequencing reads. (Sheet 1) Accession numbers for protein-coding sequences used in Figure 3. Summary of the 454sequencing data. Detailed analyses of the small cryptic plasmids.
(XLSX)

Table S2 Sequencing data and assembly statistics.
(DOCX)

Table S3 Characteristics of identified small cryptic plasmids.
(DOCX)

Table S4 Hits from pfam searches. Table of sequencing reads and their corresponding pfam hit, E-value, and pfam clan. Description of pfam domains found in all isolates.
(XLSX)

Table S5 Protein domains identified in all isolates.
(DOCX)
