# Supplementary material for: Plasmidome-Analysis of ESBL-Producing Escherichia coli Using Conventional Typing and High-Throughput Sequencing
Source: PLoS One. 2013 Jun 13;8(6):e65793. doi: 10.1371/journal.pone.0065793 (PMC3681856; doi:10.1371/journal.pone.0065793)
Supplement: Table S2 — Sequencing data and assembly statistics. (DOCX) [file pone.0065793.s004.docx]

## Table S2. Sequencing data and assembly statistics

| **Isolate (ECO-)** | **No. reads^a^** | **No. contigs^b^** | **No. contigs > 5 kb** | **N50 (bp)^c^** |
| --- | --- | --- | --- | --- |
| 005 | 15,090 (238 bp) | 133 (319 bp) | 9 | 8,071 |
| 008 | 28,222 (244 bp) | 347 (402 bp) | 2 | 711 |
| 019 | 38,489 (237 bp) | 1,803 (342 bp) | 6 | 413 |
| 029 | 38,607 (236 bp) | 976 (332 bp) | 6 | 438 |
| 033 | 38,122 (241 bp) | 2,676 (356 bp) | 12 | 420 |
| 071 | 26,233 (238 bp) | 283 (320 bp) | 2 | 521 |
| 135 | 30,189 (240 bp) | 609 (338 bp) | 12 | 2,012 |
| 147 | 44,953 (238 bp) | 156 (345 bp) | 19 | 16,134 |
| 163 | 26,460 (240 bp) | 140 (304 bp) | 7 | 27,905 |
| 299 | 25,296 (236 bp) | 112 (310 bp) | 14 | 20,061 |

^a^ The number of Roche 454-pyrosequencing reads. The median read length is specified within parenthesis.

^b^ Assembled sequencing reads. The median contig length is specified within parenthesis.

^c^ Half of all bases reside in contigs of this size or longer.
