# Supplementary material for: Plasmidome-Analysis of ESBL-Producing Escherichia coli Using Conventional Typing and High-Throughput Sequencing
Source: PLoS One. 2013 Jun 13;8(6):e65793. doi: 10.1371/journal.pone.0065793 (PMC3681856; doi:10.1371/journal.pone.0065793)
Supplement: Table S3 — Characteristics of identified small cryptic plasmids. (DOCX) [file pone.0065793.s005.docx]

## Table S3. Characteristics of identified small cryptic plasmids

| **Isolate (ECO-)** | **Plasmid** | **454 assembly size (nt)** | **Average coverage** | **Replicon type** | **Best match** | **Resistance genes** |
| --- | --- | --- | --- | --- | --- | --- |
| 008 | pEC08-6 | 5165 | 200 | Replicase superfamily | pCE10B (CP003036) 100% covered, 98.5% identity | - |
|  | pEC08-5 | 4593 | 296 | ColE1 | pSN11/00 (GQ470395) 69% covered, 100% identity | - |
|  | pEC08-4 | 4082 | 72 | rep_3 superfamily | pSE11-6 (AP009246) 100% covered, 99.8% identity | - |
|  | pEC08-3 | 4072 | 60 | rep_3 superfamily | E. coli NA114 chromosomal region (CP002797) 100% covered, 99.9% identity | - |
|  | pEC08-2 | 2101 | 353 | rep_1 superfamily | pSS046 (CP000643) 100% covered, 100% identity | - |
|  | pEC08-1 | 1780 | 153 | rep_1 superfamily | pB (AY178821) 65% covered, 94.6% identity | - |
| 019 | pEC19-1 | 4860 | 678 | ColE1 | pUUH239.1 (CP002473) 56% covered, 98.5% identity | - |
| 029 | pEC29-1 | 4082 | 45 | rep_3 superfamily | pSE11-6 (AP009246) 100% covered, 99.8% identity | - |
| 033 | pEC33-1 | 2101 | 1930 | rep_1 superfamily | pSS046 (CP000643) 100% covered, 99.8% identity | - |
| 071 | pEC71-2 | 4315 | 338 | ColE1 | pEC886 (HQ659758) 47% covered, 99.8% identity | - |
|  | pEC71-1 | 2026 | 82 | Rep protein (unknown family) | No match | - |
| 135 | pEC135-1 | 2101 | 584 | rep_1 superfamily | pSS046 (CP000643) 100% covered, 99.99% identity | - |
| 147 | pEC147-4 | 4071 | 67 | rep_3 superfamily | E. coli NA114 chromosomal region (CP002797) 100% covered, 99.9% identity | - |
|  | pEC147-3 | 3904 | 57 | Replicase superfamily | pCROD3 (FN543505) 100% covered, 96.4% identity | - |
|  | pEC147-2 | 2088 | 636 | rep_1 superfamily | pSS046 (CP000643) 100% covered, 99.9% identity | - |
|  | pEC147-1 | 1565 | 1076 | Rep protein (unknown family) | pSERB2 (DQ269444) 64% covered, 100% identity | - |
| 163 | pEC163-1 | 2089 | 532 | rep_1 superfamily | pSS046 (CP000643) 100% covered, 99.9% identity | - |
| 299 | pEC299-4 | 6199 | 183 | Rep protein (unknown family) | p62 (FN822747) 99.6% covered, 99.7% identity | sul2, strA+B |
|  | pEC299-3 | 5165 | 206 | Replicase superfamily | pCE10B (CP003036) 100% covered, 99.3% identity | - |
|  | pEC299-2 | 4088 | 60 | rep_3 superfamily | pSE11-6 (AP009246) 95% covered, 98.2% identity | - |
|  | pEC299-1 | 1546 | 267 | Rep protein (unknown family) | pJD8 (HQ328804) 100% covered, 99.5% identity | - |
