# Supplementary material for: Plasmidome-Analysis of ESBL-Producing Escherichia coli Using Conventional Typing and High-Throughput Sequencing
Source: PLoS One. 2013 Jun 13;8(6):e65793. doi: 10.1371/journal.pone.0065793 (PMC3681856; doi:10.1371/journal.pone.0065793)
Supplement: Table S5 — Protein domains identified in all isolates. (DOCX) [file pone.0065793.s007.docx]

## Table S4. Protein domains identified in all isolates

| **Pfam domain** | **# reads^a^** | **Description** |
| --- | --- | --- |
| Phage_integrase | 77.5 | Cleave DNA substrates. |
| IncFII_repA | 67.5 | Essential for plasmid replication. |
| DUF1380 | 61.5 | Unknown function. |
| PsiA | 61 | Possibly SOS signal generation. |
| Beta-lactamase2 | 55 | Antibiotics resistance. |
| RepB-RCR_reg | 50.5 | Replication regulatory protein. |
| N6_N4_Mtase | 49 | DNA methyltransferase. |
| SSB | 46.5 | Single-strand binding protein |
| Antirestrict | 46 | Inhibits restriction by members of the three families of type I systems. |
| ParBc | 45 | Unknown function. |
| DUF932 | 42 | Unknown function. |
| DUF2726 | 35 | Unknown function. |
| rve | 32.5 | Retroviral integrase. |
| DUF1472 | 29.5 | Unknown function. |
| Methyltransf_26 | 25.5 | Methyltransferase domain. |
| RepA1_leader | 25 | Replication. |
| HTH_7 | 19 | DNA binding. |
| HTH_21 | 17.5 | DNA binding. |

^a^ Median number sequencing reads across all isolates.
